# Retraction Note: The economic commitment of climate change

**DOI:** 10.1038/s41586-025-09726-0

**Published:** 2025-12-03

**Authors:** Maximilian Kotz, Anders Levermann, Leonie Wenz

**Affiliations:** 1 Research Domain IV, Research Domain IV, Potsdam Institute for Climate Impact Research, Potsdam, Germany; 2 Institute of Physics, Potsdam University, Potsdam, Germany; 3 Mercator Research Institute on Global Commons and Climate Change, Berlin, Germany

**Keywords:** Projection and prediction, Interdisciplinary studies, Economics, Environmental health, Environmental economics

Retraction to: *Nature* 10.1038/s41586-024-07219-0 Published online 17 April 2024

The authors have retracted this paper for the following reasons: post-publication, the results were found to be sensitive to the removal of one country, Uzbekistan, where inaccuracies were noted in the underlying economic data for the period 1995–1999. Furthermore, spatial auto-correlation was argued to be relevant for the uncertainty ranges. The authors corrected the data from Uzbekistan for 1995–1999 and controlled for data source transitions and higher-order trends as present in the Uzbekistan data. They also accounted for spatial auto-correlation. These changes led to discrepancies in the estimates for climate damages by mid-century, with an increased uncertainty range (from 11–29% to 6–31%) and a lower probability of damages diverging across emission scenarios by 2050 (from 99% to 90%).

The authors acknowledge that these changes are too substantial for a correction, leading to the retraction of the paper. An updated version of the paper with these changes, which has yet to undergo peer review, is publicly available with continued open access to its data and methodology (10.5281/zenodo.15984134). The authors intend to submit a revised version of the paper for peer review. If and when published, this retraction note will be updated to include a link to the new publication. The authors appreciate the corrective role of the global scientific community and thank Thomas Bearpark, Dylan Hogan, Solomon Hsiang and Christof Schötz for bringing these issues to their attention. All authors agree to this retraction.

